# One stage transanal versus one stage laparoscopic-assisted transanal endorectal pull-through in managing Hirschsprung’s disease in pediatric age group; a retrospective study

**DOI:** 10.1186/s12893-025-02768-1

**Published:** 2025-02-08

**Authors:** Ahmed Elrouby, Sameh Shehata, Saber Waheeb, Ahmed Khairi, Doaa AbdAl-Aziz, Baher Looka

**Affiliations:** Department of Pediatric Surgery, Faculty of Medicine, Alexandria University, Alexandria, Egypt

**Keywords:** One-stage, Soave, Transanal, Laparoscopy, Outcomes

## Abstract

**Background:**

The management of Hirschsprung’s disease has evolved from the conventional route to the minimally invasive route in one stage either from the pure transanal route or with the assistance of laparoscopy. Our study compared the surgical and functional outcomes of both approaches.

**Methods:**

Our retrospective study included 72 pediatric patients presented with Hirschsprung’s Disease to Elshatby University Hospital, 40 patients were treated by TAERPT (Group A) and 32 patients were treated by LAERPT (Group B). The two groups were compared as regards the personal data, the operative data, and the post-operative outcomes including the time of passage of stools, time of tolerating oral feeding, the duration of hospital stay, and the development of any early postoperative complications. Moreover, the frequency of defecation, constipation, enterocolitis, anastomotic stricture, and continence were assessed.

**Results:**

The age at operation was significantly lower (*p* < 0.001^*^) in patients of Group A (13.95 ± 18.18) than in patients of Group B (32.03 ± 16.20). The total operative duration was not different between the two groups, however, a significantly shorter duration of the anal part (*p* < 0.001^*^) in Group B (47.81 ± 18) than in Group A (96.50 ± 38.60) was recorded. A significantly longer colonic segment (*p* < 0.001^*^) was resected in Group A (28 ± 4.05) than in Group B (22.70 ± 8.12). The hospital stay was significantly shorter in Group B (5.78 ± 2.41) than in Group A (7.20 ± 2.78). (*p* = 0.001*) The excised segment revealed a proximal aganglionic zone in four patients denoting a missed segment; three in Group B and only one patient in Group A. There were no differences as regards the early and late follow-up parameters.

**Conclusions:**

Endorectal pull-through for the treatment of Hirschsprung’s disease could be approached either completely transanal or with the assistance of laparoscopy with nearly similar surgical and functional outcomes, however a longer operative duration with a shorter anal stage is recorded with the laparoscopic assistance. Furthermore, a shorter hospital stay could be achieved with the aid of laparoscopy.

**Trial registration:**

Protocol ID: 0306356, Registration number: NCT06419998, 20/05/2024 - Retrospectively registered

## Background

The approach in surgical treatment of HD has evolved during the last years from conventional abdominal surgery to minimal invasive surgery (MIS). MIS in this operation could be completed either from the pure transanal route or with the addition of laparoscopic dissection. MIS reduces the laparotomy-associated co-morbidities such as post-operative pain, which increases the need for post-operative analgesia. Furthermore, using a pure transanal approach could minimize the post-operative pain to a further extent as the incision and dissection are started above the anoderm where there are no somatic nerve fibers. Additionally, the traditional conventional surgery usually prolongs the duration of hospital stay which increases the use of high-level analgesics and requires repeated dressings [1, 2]. 

Another achievement was the transition from a multi-staged procedure to a single-stage operation without preliminary diverting stoma; this was described for the 1st time in the 1980s [3, 4]. This was followed a few years later by the 1st description of one-stage laparoscopic-assisted endorectal pull-through (LAERPT) by Georgeson et al. in 1995 [5]. The era of one-stage laparoscopic pull-through extended later on to include both Swenson and Duhamel procedures [6, 7]. 

Later on, De La Torre–Mondragon et al. described for the 1st time a total and pure transanal endo-rectal pull-through (TAERPT) procedure for the treatment of HD in 1998 [8]. This was followed by the description of the pure transanal Swenson pull-through procedure; however, the pure transanal Duhamel pull-through is not feasible [9]. 

Each of the previously described approaches has its advantages and drawbacks. The pure TAERPT approach has the advantage of shorter operative duration, however with poor localization of the proximal transition zone before the beginning of the procedure; this could increase the rate of conversion into an open laparotomy in case of long segment HD. Another point is that the prolonged anal sphincter stretch time can affect greatly the post-operative continence level [10]. 

The utilization of laparoscopy for proximal colonic dissection has the advantage of accurate localization of the transition zone at the beginning of the procedure and consequently minimizing the anal stretch time and hence improving the postoperative continence results [11]. 

Several meta-analyses were conducted to describe the follow-up results of TAERPT and LAERPT without clear identification of the superiority of one over the other. A meta-analysis published in 2015 including 405 patients from 2107 studies comparing both procedures revealed non-significant differences as regards the postoperative results and recommended longer periods of follow-up on a wider scale of patients to pick up any advantage of one procedure over the other [11]. 

Another meta-analysis studying the long-term follow-up results after LAERPT revealed that more than one-third of the studied patients complained of post-operative bowel-associated morbidities as enterocolitis, and/or soiling. Several patients in this study required a redo surgery and consequently, they recommended a comparative study between pure TAERPT and LAERPT [12]. 

As several meta-analyses did not prove the superiority of any studied procedure over the other, our work aims to compare the early and late postoperative outcomes of the TAERPT and LAERPT in the management of HD in the pediatric age group in the aspect of improving the functional result and minimizing the post-operative complications.

## Methods

Our retrospective study included 72 pediatric patients presented with HD at Elshatby University Hospital. They were divided into two groups; Group A included 40 patients who were treated by TAERPT and Group B included 32 patients who were treated by LAERPT. The hospital records of the studied patients were reviewed and the demographic data including age at operation and at follow up were recorded.

The operative data including the operative duration, blood transfusion, and the need for insertion of a rectal tube at the end of the procedure were reviewed. All the data regarding the excised specimen as its length (total length, length of the narrow segment, and the dilated segment), and the result of its histopathological examination (presence or absence of missed segment) were recorded and tabulated.

The total postoperative follow-up period was documented and the early follow-up parameters including the time to passage of stools, time of tolerating oral feeding, duration of hospital stay, and the development of any early postoperative complications were also documented.

The late follow-up parameters including the frequency of defecation, constipation, enterocolitis, abdominal distension, anastomotic stricture, and continence were recorded and documented. Postoperative continence was tested using the Abbreviated Baylor Social Continence Scale based on 6 questions. Scores ranged from 0 to 24 and lower scores reflected better social continence [13]. The two groups were compared according to all studied parameters according to our statistical analysis methods.

### Statistical analysis

The data of our study was fed, tabulated, and described using IBM SPSS software package version 20.0. (Armonk, NY: IBM Corp). The qualitative data were shown using numbers and percentages, however, the quantitative data were shown using range (minimum and maximum), mean, standard deviation, and median (IQR). The categorical variables were compared using the Chi-square test. If the expected count is less than 5 in more than 20% of cells; Fisher’s Exact was used in comparison. On the other hand; if the quantitative data was abnormally distributed, the Mann-Whitney test was used. The level of significance was evaluated at the level of 5%.

## Results

Out of the 72 patients; 59 patients (81.9%) were females and 13 were males (18 > 1%) with a female: male ratio of 4.5:1. The youngest patient in our study was operated on at two months old and the oldest patient was 8 years old, with a significantly younger age of patients in Group A (13.95 ± 18.18 months) than in Group B (32.03 ± 16.20 months). **(Mann Whitney test;** U = 327.00^*^, *p* < 0.001^*^)

The overall operative duration was not different between the two groups, however, the laparoscopic dissection in Group B reduced the duration of the transanal step, and hence the anal stretch time to be significantly shorter than in Group A. (*p* < 0.001^*^). (Table 1)

As regards the length of the resected colonic segment, it was significantly shorter in patients of Group B than in those belonging to Group A; (*p* < 0.001^*^). A similar finding was noticed while measuring the length of the dilated part, which was significantly longer in Group A than in Group B (*p* < 0.001^*^). On the other hand, the length of the narrow aganglionic segment was nearly similar in both groups. (Table 1)

One-quarter of the operated patients received intraoperative blood transfusion; of them, 12 patients (16.67%) were in Group A and only six patients in Group B (8.3%) without significant difference (Mann Whitney test, U = 1.200, *p* = 0.273). A rectal tube was inserted trans-anastomotic at the end of the procedure in all patients to divert stools from the anastomotic line for some period, helping a protected anastomotic healing.

**Table 1:** Comparison of the operative data between the two studied groups

	Group A (*n* = 40)	Group B (*n* = 32)	Test of Significance	*P*
**Operative duration (min)**				
•** Total**				
Mean ± SD.	96.50 ± 38.60	107.4 ± 34.71	U = 521.00	0.174
Median (Min. – Max.)	90 (50–180)	100 (60–210)		
• **Lap time in lap assisted.**				
Mean ± SD.	–	59.59 ± 22.74	–	–
Median (Min. – Max.)	–	55 (30–120)		
• **TAPT time**				
Mean ± SD.	96.50 ± 38.60	47.81 ± 18	U = 159.50^*^	< 0.001^*^
Median (Min. – Max.)	90 (50–180)	45 (20–90)		

	**Group A** **(***n* = **40)**	**Group B** **(***n* = **32)**	**Test of Significance**	***p***
**Length of the resected segment (cm)**				
• **Total**				
Mean ± SD.	28 ± 4.05	22.70 ± 8.12	U = 308.00	< 0.001^*^
Median (Min. – Max.)	30 (20–35)	20 (11–45)		
• **The dilated segment**				
Mean ± SD.	20.87 ± 4.22	11.16 ± 4.63	U = 97.00^*^	< 0.001^*^
Median (Min. – Max.)	20 (15–30)	10 (2–22)		
• **The narrow segment**				
Mean ± SD.	7.13 ± 2.50	7.50 ± 3.94	U = 606.00	0.689
Median (Min. – Max.)	5 (5–10)	6 (2–18)		

**SD**: Standard deviation, **U**: Mann Whitney test, ***p***: *p*-value for comparing between the studied groups *: Statistically significant at *p* ≤ 0.05

Regarding the immediate post-operative follow-up parameters; most of our studied patients passed their first bowel motion during the first two post-operative days. Following their first motion; they started oral intake from the 2nd postoperative day, with a significantly shorter period of hospital stay in Group B than in Group A (*p* = 0.001*). (Table 2)

Immediately following the procedure, the excised colonic segment was examined by the pathologists for the presence of ganglion cells in its proximal end and aganglionosis in its distal end to confirm both the diagnosis and the successful resection. Proximal normal ganglia were detected in 68 patients (94%), while aganglionosis was found in the remaining four patients (6%) denoting missed proximal segment of HD; three belonging to Group B and one belonging to Group A without significant difference. (**Chi-square test**, χ^2^ = 1.601, ^FE^*p*= 0.317)

**Table 2:** Immediate postoperative follow-up parameters

	Group A (*n* = 40)	Group B (*n* = 32)	Test of Significance	*p*
**1st stool (day)**				
Mean ± SD.	1.95 ± 1.45	1.78 ± 1.41	U = 568.00	0.364
Median (Min. – Max.)	2 (1–7)	1 (1–8)		
**Oral tolerance (day)**				
Mean ± SD.	2.95 ± 1.85	2.81 ± 2.02	U = 553.00	0.262
Median (Min. – Max.)	2.50 (2–10)	2 (2–12)		
**Hospital stay (day)**				
Mean ± SD.	7.20 ± 2.78	5.78 ± 2.41	U = 388.0	0.001^*^
Median (Min. – Max.)	6.50 (5–15)	5 (4–15)		

**SD**: Standard deviation, **U**: Mann Whitney test, **χ**^**2**^: Chi-square test, **FE**: Fisher Exact, ***p***: *p*-value for comparing between the studied groups *: Statistically significant at *p* ≤ 0.05

There was no significant difference as regards the rate of early postoperative complications between the two groups. Ten patients complained of early complications, three patients in Group A (5%) and seven patients in Group B (21%).

During their hospital stay, two patients developed postoperative intestinal obstruction (2.8%); one in each group. They presented with persistent bilious vomiting, severe abdominal distension, non-passage of stool, and air-fluid levels in PXR abdomen standing. After a failed trial of conservative treatment; abdominal exploration was done revealing a twisted colon in the patient of Group A and massive adhesions in the patient of Group B and a divided right transverse colostomy was done in the two patients.

Gradual improvement was noticed in the patient of Group A, and he was discharged from the hospital, however, the patient of Group B deteriorated rapidly and died later on from severe sepsis.

Another major finding during the period of follow-up was the development of signs of peritoneal irritation with evolving sepsis and air under diaphragm in the PXR abdomen standing in two patients, one in each group. Abdominal exploration was immediately done, revealing anastomotic leakage in both patients and a divided double loop right transverse colostomy was done with gradual improvement in the two patients.

Early postoperative enterocolitis was reported in five patients; all of them were in Group B without improvement with medical treatment. Clinical examination revealed a severe constriction ring in two patients, and a terminal descending colostomy was done with a distal Hartman’s pouch. The other three patients have a missed segment of aganglionosis as proved by post-operative barium enema and rectal biopsy. One patient developed severe sepsis and deteriorated rapidly, so a diverting colostomy was done. The other two patients have been stabilized at first and redo LAERPT was done in one patient and redo abdominal Swenson was done in the other patient.

The last patient who developed early post-operative complications was a female patient in Group B. She complained of a recto-vaginal fistula which did not respond to Seton insertion and a simple loop transverse colostomy was done This was followed three months later by limited posterior sagittal anorectoplasty and the patient showed better wound healing.

The late follow-up of our studied patients was continued for one to three and half post-operative years, with a median age at follow-up of one and half years. The follow-up parameters included constipation, anastomotic stricture, enterocolitis, soiling, and incontinence without showing any significant difference between the two studied groups. (Table 3)

**Table 3:** Delayed post-operative follow-up parameters

	Group A (*n* = 40)	Group B (*n* = 32)	Test of significance
**Constipation (5; 6.9%)**	**2 (5%)**	**3 (9.4%)**	**Fisher Exact test**, χ^2^ = 0.527, ^FE^*p*= 0.6498
**Anastomotic stricture (6; 8.3%)**	**4 (10%)**	**2 (6.3%)**	**Chi-square test**, χ^2^ = 0.327, ^FE^*p*= 0.686
**Enterocolitis (14**,** 19.4%)**	**6 (15%)**	**8 (25%)**	**Chi-square test**, χ^2^ = 1.135, ^FE^*p*= 0.287
**Incontinence (3**,** 4.2%)**	**1 (2.5%)**	**2 (6.3%)**	**Fisher Exact test**, χ^2^ = 0.626, ^FE^*p*= 0.581
**Soiling (4**,** 5.5%)**	**2 (5%)**	**2 (6.25%)**	**Fisher Exact test**, χ^2^ = 0.053, ^FE^*p*= 1.000

## Discussion

Georgeson reported the first laparoscopic-assisted pull-through and De La Torre reported the first pure transanal pull-through; both of them preferred the endorectal Soave pull-through procedure because of its complete protection of the pelvic structures during dissection [14]. Although these procedures became widely used since they were first introduced, there was no clear consensus on the superiority of any of them over the other; so we conducted this study to compare both approaches as regards the operative data as well as the post-operative follow-up parameters to get any significant difference in the outcome between the two procedures.

The age of the studied patients at operation was significantly younger in patients treated by TAERPT mostly due to our developing curve of LAERPT procedure which imposed the selection of patients with older age. A narrower range of age (4–7 months) was observed by Ebrahim A. et al., in their similar comparative study, however without significant difference between the two studied groups [15]. 

Generally speaking, the development of the transanal approach either in pure form or with the assistance of laparoscopy reduced the operative duration greatly when compared to the conventional methods [16]. 

The operative duration was not different between the two groups in our study; on the other hand, a significantly longer operative duration was observed by Emad Y et al. in their comparative study being (160–210 min) in patients treated by LAERPT and (95–140 min) in patients treated by TAERPT [17]. The shorter operative duration in patients treated by TAERPT than in those treated by LAERPT was explained by many surgeons by the time used in accessing the abdomen and adjusting the ergonomics [2]. De la Torre L et al. added that this could be attributed to the tendency to select uncomplicated patients with short segment HD for the TAERPT procedure [18]. 

The total length of the resected bowel segment was significantly longer in patients who were treated by TAERPT than in those who were treated by LAERPT; this finding added a lot to the advantage of using laparoscopy in abdominal dissection as laparoscopy can define the level of normal colon at which the resection could be done to avoid unnecessary excision of an extra-segment which can happen in the pure transanal approach [15]. 

The total length of the excised colonic segment in our study ranged from 11 to 45 cm, this was longer than the measured length in another similar study conducted by Isa MM et al. in which the average length of the resected segment was about 18.63 cm ranging from 7 to 25 cm [19]. 

There was no significant difference in the rate of intraoperative blood transfusion between the two techniques. On the other hand, Ebrahim A. et al. reported in their study a significantly lesser blood loss in LAERPT than in TAERPT [15]. 

The time to return of bowel habits and hence the start of oral feeding were almost similar in the two groups. Cantone N et al. and Ebrahim A. et al. reported in their studies a faster return of bowel habits [15, 20]. The variation in starting oral intake between both groups in different studies could be attributed to the difference in the policy of each institute in starting oral intake whether routinely on the 1st post-operative day, immediately after the return of notable intestinal peristalsis or only after the passage of flatus and/or stools.

Furthermore, the postoperative hospital stay was significantly shorter in patients who were treated by LAERPT than in those who were treated by TAERPT. This observation could reduce greatly the hospital cost as well as the possibility of post-operative hospital-acquired infection. On the other hand, Ebrahim A. et al. reported a nearly similar duration of hospital stay in both groups in their study [15]. 

There was no significant difference in the rate of post-operative complications between the two studied techniques, similar to the findings of Karlsen RA. et al. and Emad Y. et al. in their studies [10, 16]. 

One of the postoperative complications that developed in our study was intestinal obstruction which developed in two patients (2.8%) being lower than the reported incidence in a similar comparative study conducted by Ahmed H. et al. who reported an incidence of 8–30%. The cause of the development of such complications after the pull-through procedure was explained in the literature by anastomotic problems and/or missed aganglionic segment [20]. 

Leakage from the anastomotic line developed similarly in both groups without significant difference, this finding was also recorded by Karlsen RA. et al. who reported a non-significant difference between TAERPT and LAERPT as regards anastomotic leakage (1.3–8.0%). Karlsen RA. et al. also clarified the fact that although laparoscopic utilization could identify the proximal vascularity, a local hematoma with a super added infection can result in leakage in such a situation [10]. Another explanation of such complications after the pull-through procedure was stated by Peng C-H. et al. who described that extensive local devascularization can result in local areas of necrosis resulting directly into anastomotic leakage [21]. 

There were two patients with twisted colon in the current study which could happen after pull-through when done in pure transanal form due to the blind dissection as described by Ebrahim A. et al. [15] However, Karlsen RA. et al. reported in their study that this could also happen after LAERPT despite the clear identification of the anatomy and vascularity of the pulled colon [10]. 

The patients in the current study were followed up for three and half years for the frequency of defecation, constipation, distension, enterocolitis, and continence without any significant difference between the two studied groups. Similar findings were reported by Cantone N. et al. and Karlsen RA. et al. in their studies [10, 22]. 

During the post-operative follow-up visits, digital rectal examination revealed anastomotic stricture in 10% of the patients belonging to the group treated by TAERPT and 6.3% of patients of the group treated by LAERPT; this difference was not significant similar to the findings of Ebrahim A. et al. and Karlsen et al. [10, 15] The most reasonable explanation of such complication is the circular line of anastomosis which could be avoided by oblique anastomosis and usually improves by regular anal dilatation [23]. 

Post-operative constipation was recorded in our study in five patients who responded well to laxatives with nearly similar rates in the two studied groups; this is similar to the findings of Ebrahim A. et al. [15] Keshtgar et al. attributed that to the high anal resting pressure in association with weak rectal motility [24]. 

A nearly similar incidence of post-operative enterocolitis was noticed in both groups similar to the findings of Ebrahim A. et al.; all of them were managed by regular rectal washouts, oral metronidazole, and regular transanal dilatations with acceptable and gradual improvement [15]. 

Although there was a significantly shorter period of anal stretch time in patients treated by LAERPT, there was no significant difference as regards the post-operative continence level. Patients with post-operative incontinence were referred to pelvic floor physiotherapists, and they showed gradual but slow improvement. This is similar to the findings of Karlsen RA. et al. in their comparative study between the two techniques in 2022 [10]. Generally speaking, A. Elrouby et al. concluded in their study that the Soave procedure was usually not associated with post-operative incontinence, whether done abdominal or transanal [25]. A very low level of anal dissection and/or prolonged anal stretch time may be a good explanation for the post-operative incontinence developing in such patients [26]. 

Patients who developed post-operative soiling were nearly the same in both groups, similar to the findings of Emad Y et al. in their study [16]. Regular bowel evacuation in association with a low-residue diet and loperamide reduced gradually the frequency of such complications. A higher incidence was noted in another study conducted by A Elrouby et al. which revealed temporary post-operative soiling after Soave endorectal pull-through operation in 21 patients (13%) [25]. This complication was explained in the literature by the change in stool frequency and consistency, which could develop postoperatively and usually improves spontaneously with time [27]. 

One of the patients belonging to Group B died from severe sepsis following intestinal obstruction. In a similar study, Ebrahim A. et al. reported one case who died due to severe sepsis and chest infection. There was no significant difference in the death rate between the two studied groups [15]. 

## Conclusions

Treatment of HD in the pediatric age group with endorectal dissection could be approached either completely transanal or with the assistance of laparoscopy. The laparoscopic assistance can shorten the duration of the anal stretch period and also reduces the excision of an unnecessarily longer colonic segment. Moreover, a shorter hospital stay was recorded with the aid of laparoscopy. However, the overall follow-up of either technique revealed no difference in the surgical or functional outcomes.

## Limitations of the study

Although there were no differences as regards the postoperative surgical & functional outcomes between either technique with nearly similar rates of complications, a more reliable conclusion about the superiority of selecting one of either technique would be available if a further study could be planned over a wider range of patients with a longer period of follow-up. A clear pre-operative clarification of the level of aganglionosis was a limitation in our study. This could be determined clearly in future studies to avoid affecting the results and also to decide the usage of either technique.

## Data Availability

The datasets generated and/or analyzed during the current study are not publicly available due to protection of patients’ privacy but are available from the corresponding author on reasonable request.

## Abbreviations

HD: Hirschsprung’s disease
TAERPT: Pure transanal endorectal pull-through
LAERPT: Laparoscopic-assisted endorectal pull-through
Lap: Laparoscopic
PXR: Plain X-Ray
Min: Minutes
SD: Standard deviation
U: Mann whitney test
χ2: Chi-square test
FE: Fisher Exact
p: *p*-value for comparing between the studied groups
*: Statistically significant at *p* ≤ 0.05
